# Body Weight, Body Composition, Nutrition, and Sex in Gastric Cancer: Epidemiological Associations and Surgical Outcomes—A Narrative Review

**DOI:** 10.3390/jcm15197517

**Published:** 2026-09-27

**Authors:** Claus Schildberg, Carl Meißner, Elisa Gaede, Aristotelis Perrakis, Karsten Ridwelski, René Mantke, Frank Meyer

**Affiliations:** 1 Department of General and Visceral Surgery, University Hospital Brandenburg an der Havel, Brandenburg Medical School Theodor Fontane, Hochstraße 29, 14770 Brandenburg an der Havel, Germany; 2 Faculty of Health Sciences Brandenburg, Brandenburg Medical School Theodor Fontane, Fehrbelliner Str. 38, 16816 Neuruppin, Germany; 3 Medical Care Center “Im Altstadtquartier” GmbH, Max-Otten-Straße 14, 39104 Magdeburg, Germany; 4 AN-Institut für Qualitätssicherung in der operativen Medizin gGmbH, Otto-von-Guericke University Magdeburg, Leipziger Str. 44, 39120 Magdeburg, Germany; 5 Department of General, Visceral, Vascular and Transplant Surgery, Otto-von-Guericke University Magdeburg, University Hospital Magdeburg, Leipziger Str. 44, 39120 Magdeburg, Germany; 6 Department of Visceral, Vascular and Acute Surgery, Klinikum Magdeburg gGmbH, Birkenallee 34, 39130 Magdeburg, Germany

**Keywords:** gastric cancer, body mass index, obesity, body composition, nutrition, sarcopenia, visceral adiposity, sex differences, gastrectomy

## Abstract

Gastric cancer remains a major cause of cancer-related mortality worldwide, while the roles of excess body weight, body composition, nutrition, and biological sex remain incompletely defined. This narrative review separately evaluates (i) epidemiological associations with gastric cancer risk, with explicit attention to cardia versus non-cardia tumors and *Helicobacter pylori* as a major confounder, and (ii) perioperative and long-term outcomes after gastrectomy, including postoperative complications, treatment completion, recurrence, and survival. BMI, visceral adiposity, skeletal-muscle mass, and sarcopenic obesity are treated as distinct, non-interchangeable phenotypes. Available studies are heterogeneous and partly conflicting. Prediagnostic weight loss is particularly important because cancer-related weight loss and cachexia can lower BMI at diagnosis, creating reverse causality in prognostic analyses. Visceral adiposity and sarcopenic obesity may provide clinically relevant information beyond BMI alone. Sex-related differences in adipose distribution and hormonal exposure may also modify risk and outcome, but observational associations do not establish causality. Overall, BMI alone is an insufficient marker of risk; prospective studies should integrate premorbid weight, longitudinal weight change, body composition, nutritional status, tumor subsite, *H. pylori* status, and sex-specific analyses.

## 1. Introduction

Metabolic disorders and related conditions, including excessive caloric intake, overweight, obesity, fatty liver disease, and diabetes mellitus, represent major public health challenges because of their association with secondary complications such as cardiovascular, metabolic, and malignant diseases.

Increasing evidence indicates that the relationships between diet, body weight, obesity, and cancer development—particularly with regard to sex-specific aspects—have become an important focus of medical research. Numerous studies have demonstrated that both dietary habits and body weight are significant determinants of cancer risk. Obesity, now recognized worldwide as one of the most prevalent chronic diseases, is increasingly regarded as a major risk factor for the development of various malignancies [1].

Over the past decades, substantial evidence has shown that overweight and obesity contribute to carcinogenesis through several pathophysiological mechanisms. In particular, increased body fat has been associated with alterations in hormonal regulation, chronic inflammation, and insulin resistance, all of which may promote cancer development [2,3].

Diet also plays a crucial role. Diets rich in saturated fats, refined carbohydrates, and sugar not only promote obesity but may also directly increase cancer risk. In contrast, a balanced diet rich in dietary fiber, plant-based foods, and healthy fats appears to exert protective effects against both obesity and cancer development [4].

The role of obesity as a risk factor for cancer and the importance of dietary prevention are supported by numerous international organizations and scientific studies. The World Health Organization (WHO) has emphasized the close relationship between overweight, obesity, and the incidence of several malignancies, including breast, colorectal, and kidney cancer [5]. Accordingly, there is broad scientific consensus that obesity prevention and healthy nutrition represent effective strategies for reducing both chronic disease burden and cancer risk.

With specific regard to gastric cancer, overweight and obesity have also been identified as relevant risk factors. Therefore, these factors deserve more systematic consideration in studies investigating surgical treatment and clinical outcomes.

Besides colorectal cancer, gastric cancer remains one of the most common malignant diseases encountered in abdominal surgery. Consequently, this tumor entity should be included in a more comprehensive evaluation of obesity- and nutrition-related risk factors.

The central question of this narrative review was whether anthropometric and nutritional phenotypes provide clinically meaningful information on gastric cancer risk and outcomes beyond BMI alone. To address this question without conflating etiological and prognostic associations, the evidence was organized into two complementary domains. First, we evaluated epidemiological associations of body weight, adiposity, nutritional factors, and biological sex with gastric cancer risk, explicitly distinguishing cardia from non-cardia tumors where data were available and considering *H. pylori* as a major etiological factor and potential confounder. Second, we assessed perioperative and long-term outcomes after gastric cancer treatment, including postoperative morbidity, treatment completion, recurrence, and survival, with particular attention to BMI, visceral adiposity, skeletal-muscle mass, sarcopenic obesity, nutritional status, and prediagnostic weight loss. Molecular mechanisms are discussed as biological context rather than as a separate causal evidence domain.

## 2. Materials and Methods

This narrative review was based on a structured PubMed/MEDLINE search of literature addressing body weight, body composition, nutrition, biological sex, *H. pylori*, and gastric cancer. The search was organized around two prespecified evidence domains: (1) epidemiological risk and tumor development and (2) perioperative and long-term outcomes after gastric cancer treatment.

PubMed/MEDLINE was searched from database inception to 8 August 2026. The representative core Boolean strategy was: (“gastric cancer” OR “stomach neoplasm*” OR “gastric adenocarcinoma”) AND (“body mass index” OR BMI OR overweight OR obesity OR “body composition” OR adiposity OR “visceral fat” OR “skeletal muscle” OR sarcopenia OR “sarcopenic obesity” OR nutrition OR malnutrition OR cachexia OR “weight loss” OR “prediagnostic weight loss” OR “*Helicobacter pylori*” OR sex OR gender OR hormone*) AND (risk OR epidemiology OR incidence OR cardia OR non-cardia OR gastrectomy OR surgery OR perioperative OR postoperative OR complication* OR recurrence OR survival OR prognosis). Targeted supplementary searches combined “gastric cancer” with terms for adjuvant chemotherapy, ERAS, nutritional support, clinical guidelines, microRNA/miRNA, and sex-specific or hormonal factors. Reference lists of relevant reviews, meta-analyses, guidelines, and key primary studies were also screened for additional publications.

Peer-reviewed human studies, systematic reviews/meta-analyses, and current clinical guidelines were considered when they addressed at least one of the review domains. Gastric-cancer-specific evidence was prioritized; studies of other gastrointestinal malignancies were retained only when they provided limited contextual information for concepts not adequately characterized in gastric cancer. Studies were excluded when they were clearly unrelated to gastric cancer or the prespecified exposures/outcomes, were single case reports, or did not provide clinically interpretable information for the narrative synthesis. Titles and abstracts were screened first, followed by full-text assessment of potentially relevant reports. Because the search was conducted as a narrative rather than a systematic review, a PRISMA-style screening log was not prospectively maintained; therefore, an exact retrospective count of all records screened cannot be reported reliably. The final synthesis comprised the studies and guidelines cited in the manuscript. No formal risk-of-bias assessment, protocol registration, or quantitative meta-analysis was undertaken. Evidence was synthesized separately for epidemiological risk and for perioperative/long-term outcomes, with cardia and non-cardia gastric cancer distinguished whenever the source data allowed (Table 1).

## 3. Results

Although the incidence and mortality of gastric cancer have declined substantially over the past 50 years, it remains one of the leading causes of cancer-related death worldwide.

In Western Europe, the incidence and mortality of gastric cancer have declined substantially over recent decades. For Germany, current clinical reference information and the most recent national cancer-registry report (Krebs in Deutschland für 2021–2023, 15th edition) provide the epidemiological context [6,7]. The national cancer-registry data document 5580 new cases in women and 9040 in men in 2023. The corresponding age-standardized incidence rates (old European standard population) were 6.9 per 100,000 women and 13.6 per 100,000 men [7].

The median age at diagnosis in 2023 was 74 years in women and 71 years in men. Relative 5-year survival for the 2021–2023 period was approximately 41% in women and 36% in men [7]. These updated national data supersede older case counts and survival estimates previously reported for Germany.

In 2023, 3136 women and 4627 men died from gastric cancer in Germany [7]. Gastric cancer accounted for approximately 3.0% of all cancer deaths in women and 3.8% in men; it should therefore not be described as the third leading cause of cancer-related death among men. Current German epidemiological reporting distinguishes crude from age-standardized rates; accordingly, rate estimates in this review are explicitly identified by standardization method where applicable [7].

Despite the decline in incidence, gastric cancer remains one of the most challenging malignant diseases because of its heterogeneous global epidemiology, continuing advances in diagnostic techniques, histopathological and molecular characterization, and the increasing use of multimodal treatment strategies with both curative and palliative intent. These developments have contributed to continuous improvements in patient outcomes.

*Helicobacter pylori* infection should be considered a central etiological factor when interpreting gastric cancer epidemiology. *H. pylori* is recognized as the primary infectious cause of gastric cancer, particularly non-cardia gastric adenocarcinoma, and acts through chronic gastritis with progression to atrophy, intestinal metaplasia, dysplasia, and ultimately carcinoma in susceptible individuals. Recent evidence further supports *H. pylori* eradication as an effective strategy for reducing gastric cancer risk. Accordingly, associations between obesity, nutrition, body composition, and gastric cancer should be interpreted in the context of *H. pylori* status whenever such information is available, because failure to account for infection may contribute to residual confounding and heterogeneity between studies [8,9].

Several risk factors have been investigated. For example, preoperative osteopenia has recently been identified as an independent risk factor for non-cancer-related mortality following gastrectomy in elderly patients [10].

In general, obesity is considered an established risk factor for the development of several gastrointestinal malignancies, including colorectal and pancreatic cancer, and may also adversely affect early postoperative outcomes after oncological surgery [11,12,13,14,15,16,17,18].

The available literature reports heterogeneous and, in part, conflicting findings regarding the influence of body weight, obesity, and nutritional status on the development and surgical treatment of gastric cancer.

WHO criteria define BMI-based obesity, while gastric-cancer reviews and epidemiological reports describe obesity and other metabolic factors among the risk factors considered in gastric carcinogenesis [19,20,21,22].

Tumor subsite is particularly important when interpreting obesity-related epidemiology. A large Japanese pooled cohort analysis reported no overall association between BMI and esophageal or gastric cancer, but subgroup analyses suggested differences by tumor location, including the cardia [23]. More recent pooled evidence from the Stomach Cancer Pooling Project found obesity associated with higher cardia gastric cancer risk, whereas no association was observed for non-cardia cancer. This contrasts with the 2023 meta-analysis by Azizi et al., which found higher overall gastric cancer risk with overweight and obesity but no statistically significant cardia/non-cardia difference [24,25]. Thus, the direction of evidence is more consistent for cardia cancer, but heterogeneity remains and subsite-specific analyses are essential.

German guideline and cancer-registry sources provide national clinical and epidemiological context for gastric cancer [26,27]. Cancer-associated cachexia and disease-related malnutrition may coexist with overweight or obesity and should therefore be assessed independently of BMI.

Several biological mechanisms may link excess adiposity and metabolic dysfunction with gastric carcinogenesis, including insulin resistance and altered insulin/IGF signaling, chronic low-grade inflammation, adipokine dysregulation, oxidative stress, and changes in the local and intestinal microbiome. These pathways may interact with dietary exposures and physical inactivity and may differ according to tumor subsite and *H. pylori* status. At the molecular level, microRNAs provide an additional regulatory layer. The review suggested by the reviewer describes miR-129-5p as a regulator of digestive-cancer-related pathways and summarizes gastric-cancer-related targets including ADAM9 and HMGB1, while also discussing broader WNT and PI3K/AKT/mTOR signaling. These mechanistic observations are biologically informative but should not be interpreted as evidence that obesity acts through a single molecular pathway in gastric cancer [28].

Cancer-associated metabolic alterations should be considered independently of BMI. Contemporary clinical-nutrition guidance emphasizes that reduced intake, systemic inflammation, catabolism, and loss of skeletal muscle may occur across the BMI spectrum. Consequently, overweight or obesity does not exclude clinically relevant malnutrition, cachexia, or sarcopenia, and longitudinal weight change and nutritional status should be assessed alongside body size [29,30].

### 3.1. Definition of Obesity

According to the World Health Organization (WHO), obesity is defined as a BMI ≥ 30 kg/m^2^ and is classified into three grades of severity. Grade I obesity is defined as a BMI of 30.0–34.9 kg/m^2^, Grade II obesity as a BMI of 35.0–39.9 kg/m^2^, and Grade III obesity as a BMI ≥ 40.0 kg/m^2^. Pre-obesity is defined as a BMI between 25.0 and 29.9 kg/m^2^ [19] (Table 2).

### 3.2. Anthropometric and Body-Composition Phenotypes

BMI, visceral adipose tissue, skeletal-muscle mass, and sarcopenic obesity describe different biological phenotypes and should not be used interchangeably. BMI is a size-for-height measure and does not distinguish fat from lean tissue or indicate fat distribution. Visceral adiposity reflects the amount and distribution of intra-abdominal fat and may capture metabolic and technical surgical risk that is not represented by BMI. CT-derived skeletal-muscle measures identify muscle depletion, whereas sarcopenic obesity denotes the coexistence of excess adiposity with reduced muscle mass and/or function. Consequently, two patients with the same BMI may have markedly different adipose distribution, muscle reserves, nutritional vulnerability, and perioperative risk. Throughout this review, these measures are therefore interpreted as complementary rather than equivalent markers [31,32,33,34,35].

Bischoff reported that obese individuals exhibit alterations in the composition of the intestinal microbiota, which are associated with impaired intestinal barrier function and a higher prevalence of chronic low-grade inflammation [36]. Similar inflammatory alterations have also been described in patients with malnutrition [37].

Dhar and colleagues demonstrated that an increased BMI (>24.7 kg/m^2^ in men and >22.6 kg/m^2^ in women) was associated with a significantly higher risk of tumor recurrence (relative risk 1.85; *p* = 0.03), prolonged operative time, and an increased rate of postoperative complications following gastric cancer surgery [38].

Perioperative nutritional care should follow current surgical and oncology nutrition guidance rather than BMI alone. The updated ESPEN guideline on clinical nutrition in surgery emphasizes early oral feeding, avoidance of prolonged preoperative fasting, early initiation of nutritional therapy when nutritional risk is identified, and integration of nutrition with mobilization and prehabilitation [29]. The ESPEN cancer guideline similarly recommends systematic nutritional assessment and individualized nutritional care in patients with cancer [30]. Enteral or oral routes are preferred when feasible, whereas parenteral nutrition is reserved for patients in whom nutritional requirements cannot be met through the gastrointestinal tract. These contemporary recommendations update the older nutrition literature retained for historical context [39,40,41,42,43].

Karletshofer et al. and Spiller reported that BMI itself was not an independent prognostic factor. Instead, prognosis was primarily determined by:

Pathological TNM/UICC stage;

Histological growth pattern;

Tumor location;

Surgical treatment;

Residual tumor status [44,45].

Epidemiological evidence suggests that the association of overweight and obesity with gastric cancer is more pronounced for cardia than for non-cardia carcinoma [46].

Another important risk factor is sarcopenia and sarcopenic obesity. CT-based body-composition assessment can identify low skeletal-muscle mass and sarcopenic obesity, which have been associated with postoperative complications and adverse outcomes after gastrectomy [31,32,33]. These findings support early nutritional and functional assessment, although the reviewed studies do not by themselves prove that protein supplementation improves oncological outcomes.

An additional aspect is visceral adiposity, which appears to be more relevant than BMI in men. Lower visceral fat mass in men has been associated with poorer long-term survival, whereas reduced subcutaneous fat has been linked to worse survival in women [34].

Biological sex, exogenous or endogenous hormonal exposure, and body-fat distribution are related but distinct variables and should be analyzed separately. Biological sex encompasses chromosomal and sex-related physiological differences; hormonal exposure varies within each sex and across the life course; and visceral versus subcutaneous adipose distribution represents a body-composition phenotype that may itself differ by sex. Observational studies have reported associations between circulating sex hormones or reproductive/hormonal exposures and gastric cancer risk [47,48]. In the meta-analysis by Camargo et al., longer reproductive lifespan and hormone replacement therapy were associated with lower gastric cancer risk, whereas parity was not significantly associated with risk [48]. These associations may reflect hormonal, metabolic, behavioral, or residual confounding and should not be interpreted as evidence of a causal protective estrogen effect.

Studies in women have reported associations between adiposity and circulating sex-steroid hormone concentrations [49,50,51], while a large cohort study found that the association between obesity and gastric cancer risk differed by menopausal status [52]. Separately, CT-based studies suggest sex-specific prognostic patterns of visceral and subcutaneous adipose tissue [34]. The revised synthesis therefore distinguishes biological sex, hormonal exposure, and fat distribution rather than treating them as interchangeable explanations for sex-related differences (Table 3).

### 3.3. Prediagnostic Weight Loss and Reverse Causality

Prediagnostic weight loss requires separate consideration from BMI because gastric cancer itself can reduce food intake and induce systemic catabolism and cachexia before diagnosis. Thus, a low BMI measured at diagnosis may be a consequence of more advanced or biologically aggressive disease rather than a pre-existing exposure, creating reverse causality. In a cohort of 1330 patients undergoing curative resection, preoperative weight loss was independently associated with overall survival, particularly in stage III disease [54]. In a Veterans Health Administration cohort of upper gastrointestinal adenocarcinomas, increasing prediagnostic weight loss was associated with mortality independently of BMI, and adjustment for premorbid BMI and weight loss substantially altered the apparent BMI-survival relationship [55]. More recently, a multicenter Chinese cohort of 29,779 patients also incorporated usual BMI and prediagnostic weight loss and found that weight loss exceeding 10% of usual weight was associated with poorer prognosis [56]. These findings indicate that prognostic analyses based only on BMI at diagnosis may overestimate an apparent protective effect of overweight or obesity. Whenever possible, premorbid weight, percentage weight loss, its time course, and markers of cachexia should therefore be analyzed alongside BMI.

### 3.4. Interim Summary

The available evidence highlights the multifactorial relationship between body weight, nutritional status, body composition, and gastric cancer. Importantly, overweight or obesity does not exclude clinically relevant malnutrition or sarcopenia. Visceral and subcutaneous adipose-tissue compartments may carry prognostic information beyond BMI, and their relevance may differ by sex [34,48,49,50,51,52,53]. Accordingly, body-composition and nutritional assessment should complement BMI in perioperative risk assessment. In patients with sarcopenia or sarcopenic obesity, individualized nutritional and functional interventions may be appropriate according to clinical nutrition guidance; however, the reviewed gastric-cancer studies do not establish that protein supplementation itself improves recurrence or long-term oncological survival [29,30,31,32,33].

### 3.5. Recent Evidence

Several recently published studies have further expanded current knowledge regarding body weight, body composition, and prediagnostic weight change in gastric cancer. Their designs, effect estimates, relevance, and principal limitations are summarized in Table 4.

Azizi et al. conducted a systematic review including more than 14 million individuals and reported pooled risk ratios for gastric cancer of:

1.124 (95% CI, 0.968–1.304; I^2^ = 89.08%) in the underweight group;

1.155 (95% CI, 1.051–1.270; I^2^ = 95.18%) in the overweight group;

1.218 (95% CI, 1.070–1.386; I^2^ = 97.65%) in the obesity group.

The underweight estimate was not statistically significant because its 95% CI included 1.00, whereas overweight and obesity were associated with higher gastric cancer risk. No significant difference was observed between cardia and non-cardia gastric cancer. Meta-regression suggested stronger associations for excess body weight in women than in men and in non-Asian than Asian populations [24].

Pescari and colleagues compared two approaches for classifying obesity: body mass index (BMI) and body fat percentage (BFP), measured by bioelectrical impedance analysis (BIA), in a cohort of 1255 adults.

The authors found that age and psychological factors—including food cravings, fatigue, stress, and compulsive eating—were significant predictors of obesity when BMI was used but not when obesity was classified according to body fat percentage. These findings indicate that BMI and BFP reflect different aspects of obesity and should not be regarded as interchangeable measures [57].

Kuichanuan and co-workers proposed a revised BMI cut-off value for diagnosing obesity in young Thai adults. Their findings emphasize that BMI thresholds may vary between ethnic populations and suggest that population-specific definitions of obesity may improve risk assessment and clinical management [58].

### 3.6. Context from Other Gastrointestinal Malignancies

Evidence from colorectal and general surgical cohorts has sometimes shown higher perioperative morbidity in patients with obesity but similar or better long-term survival, giving rise to the term “obesity paradox” [18,59,60,61,62,63,64]. Because these findings arise from different tumor entities and are vulnerable to confounding by stage, comorbidity, body composition, prediagnostic weight loss, and treatment selection, they are included only as brief contextual information and should not be extrapolated directly to gastric cancer. Unpublished observations and data described only as manuscripts in preparation were removed from the revised review and are not used to support substantive conclusions.

### 3.7. BMI, Adjuvant Chemotherapy, and Survival

For patients with resectable stage II/III gastric cancer, systemic treatment is an important component of multimodal therapy. The studies summarized below suggest that BMI and nutritional status may be associated with treatment completion and survival, although these associations should not be interpreted as causal.

Tsuburaya et al. [65] performed a pooled analysis of 3527 patients enrolled in large randomized controlled trials to identify prognostic and predictive markers for adjuvant treatment following D2 lymphadenectomy. Age, in addition to tumor stage, was identified as an independent prognostic factor. Adjuvant chemotherapy improved relapse-free survival and overall survival across all TN stages; however, the benefit appeared greater in patients with T1–2 tumors than in those with T3–4 tumors. Furthermore, treatment efficacy was higher in patients with low or intermediate BMI than in those with high BMI (hazard ratios: 0.58, 0.58, and 1.05, respectively).

Peng et al. [66] retrospectively analyzed 2510 consecutive patients with stage II/III gastric cancer who underwent curative gastrectomy. A low preoperative BMI (<20.3 kg/m^2^) independently predicted incomplete perioperative adjuvant chemotherapy (<6 treatment cycles) and poorer overall survival (hazard ratio 1.317; 95% confidence interval 1.162–1.494; *p* < 0.001). Patients with both low BMI and incomplete chemotherapy had the poorest survival, whereas completion of at least six cycles was associated with improved survival in the low-BMI subgroup. These data identify low BMI as a marker of vulnerability and treatment non-completion, but they do not establish that nutritional supplementation itself improves oncological survival. The recent risk studies are summarized in Table 4; studies of BMI, chemotherapy completion, and survival are summarized separately in Table 5.

Geographic treatment context is also important when interpreting these associations. Much of the evidence linking BMI or nutritional status to chemotherapy completion after gastrectomy derives from Asian cohorts using postoperative adjuvant strategies. In Western practice, perioperative chemotherapy is widely used for locally advanced resectable gastric and gastro-oesophageal junction adenocarcinoma; for example, the German FLOT4 trial evaluated four preoperative and four postoperative cycles of FLOT and demonstrated improved overall survival compared with perioperative ECF/ECX [67]. Consequently, associations between body composition, treatment completion, and survival observed in predominantly Asian adjuvant-treatment cohorts should not be assumed to transfer directly to Western populations, in which treatment timing, duration, and regimen selection may differ. Future studies should examine these associations across geographic settings and contemporary treatment strategies.

### 3.8. Sarcopenia and Sarcopenic Obesity

Sarcopenia and sarcopenic obesity are clinically relevant in gastric cancer because BMI can mask depletion of skeletal muscle. Recent meta-analyses have strengthened the evidence that sarcopenic obesity is associated with postoperative complications and poorer overall survival after gastrectomy, while also emphasizing heterogeneous definitions and predominantly observational evidence [31,32,33,35].

Current perioperative guidance supports screening for malnutrition, sarcopenia, and frailty and initiating individualized nutritional and functional interventions when risk is identified [29,30].

Sarcopenic obesity should therefore be viewed as a body-composition phenotype rather than inferred from BMI alone. CT-derived muscle and adipose-tissue measurements can complement conventional nutritional assessment, particularly because clinically relevant muscle depletion may coexist with overweight or obesity.

Management should be individualized and may combine nutritional therapy, resistance or structured exercise, and broader prehabilitation according to clinical status. Adequate energy and protein intake are components of supportive care, but the evidence reviewed here does not demonstrate that protein supplementation per se improves recurrence, cancer-specific survival, or overall survival in gastric cancer.

### 3.9. Clinical Implications

For clinical practice, the reviewed evidence supports avoiding reliance on BMI alone. Preoperative assessment should integrate nutritional screening, recent weight change, and, where available, CT-derived measures of skeletal muscle and adipose-tissue distribution. Patients with malnutrition, sarcopenia, sarcopenic obesity, or substantial weight loss may warrant early multidisciplinary nutritional and functional support in accordance with current perioperative guidance [29,30]. These measures are intended to improve nutritional and functional status and support treatment delivery; this narrative review cannot establish a direct improvement in long-term oncological outcomes from any specific supplement or protein intervention.

### 3.10. Methodological Limitations

The evidence base is limited by heterogeneous BMI cut-offs, retrospective designs, variable adjustment for confounders, inconsistent separation of cardia and non-cardia tumors, and insufficient assessment of premorbid weight and prediagnostic weight loss. Reverse causality is a particular concern in gastric cancer because tumor-related anorexia, dysphagia, inflammation, catabolism, and cachexia can lower BMI before diagnosis; BMI measured at diagnosis may therefore partly reflect disease severity rather than antecedent adiposity. BMI, visceral adiposity, skeletal-muscle mass, and sarcopenic obesity are also methodologically distinct phenotypes, yet they are not consistently measured or modeled separately. Many studies additionally lack standardized assessment of dietary intake and physical activity. *H. pylori* status is not consistently reported or adjusted for, which is particularly important when cardia and non-cardia gastric cancers are analyzed together [54,55,56].

Analyses stratified by biological sex are frequently underpowered or absent. In addition, associations with overall survival may be confounded by stage, comorbidity, chemotherapy completion, and competing mortality. These limitations reduce comparability and help explain the partly incongruent findings.

This review itself has limitations. As a narrative review, it does not provide a systematic risk-of-bias assessment, quantitative synthesis, or a PRISMA-style study-selection process. The broad scope was chosen to integrate epidemiological, surgical, nutritional, body-composition, sex-specific biological, and *H. pylori*-related perspectives. Study selection therefore remains susceptible to selection bias, and causal conclusions cannot be drawn.

## 4. Conclusions

BMI, visceral adiposity, skeletal-muscle mass, sarcopenic obesity, and nutritional status represent complementary rather than interchangeable dimensions in gastric cancer. Epidemiological associations vary by tumor subsite, while prognostic analyses are particularly vulnerable to reverse causality from prediagnostic weight loss and cachexia. Accordingly, BMI at diagnosis should not be interpreted without premorbid weight, longitudinal weight change, and body-composition information. Future prospective studies should standardize these measures, distinguish cardia from non-cardia tumors, account for *H. pylori* status, and separate biological sex, hormonal exposure, and sex-specific fat distribution in prespecified analyses.

## Figures and Tables

**Table 1:** Structured literature-search framework and narrative synthesis approach.

Item	Description
Review design	Narrative review using a structured literature search; no formal risk-of-bias assessment or quantitative meta-analysis.
Information source	PubMed/MEDLINE; search period: database inception to 8 August 2026; supplementary reference-list screening.
Core concepts	Gastric cancer/stomach neoplasms combined with BMI, overweight/obesity, body composition/adiposity, visceral fat, skeletal muscle, sarcopenia, nutrition/malnutrition/cachexia, prediagnostic weight loss, *H. pylori*, and sex/gender.
Additional concepts	Risk/epidemiology/incidence and cardia/non-cardia subsite; gastrectomy/surgery, perioperative/postoperative complications, recurrence, survival/prognosis, chemotherapy, ERAS, nutritional support, and guidelines.
Study identification and selection	Peer-reviewed human studies, systematic reviews/meta-analyses, and current guidelines were prioritized. Titles/abstracts and relevant full texts were assessed. Single case reports and clearly off-topic studies were excluded. No prospective PRISMA log was maintained, so an exact retrospective screening count is not reported.
Evidence synthesis	Evidence was narratively synthesized in two domains: epidemiological risk versus perioperative/long-term outcomes. Cardia and non-cardia tumors were distinguished whenever source data allowed; gastric-cancer-specific evidence was prioritized.
Methodological scope	Narrative review without protocol registration, formal risk-of-bias assessment, or quantitative synthesis; causal conclusions cannot be drawn.

Legend: Methodological framework of this narrative review. BMI, body mass index; ERAS, Enhanced Recovery After Surgery; *H. pylori*, *Helicobacter pylori*. The search was structured but was not conducted as a prospectively registered systematic review; consequently, a PRISMA-style flow diagram and an exact retrospective count of all screened records are not provided.

**Table 2:** WHO classification of body mass index (BMI).

Classification	BMI (kg/m^2^)
Underweight	<18.5
Normal weight	18.5–24.9
Overweight (pre-obesity)	25.0–29.9
Obesity class I	30.0–34.9
Obesity class II	35.0–39.9
Obesity class III	≥40.0

**Legend: WHO BMI classification.**

**Table 3:** Integrated evidence framework for body weight, body composition, nutrition, sex, and *Helicobacter pylori* in gastric cancer.

Domain	Evidence Summarized in This Review	Clinical Interpretation	Main Limitation/Confounder
BMI/obesity	BMI-based evidence is heterogeneous, ranging from no independent association to adverse perioperative outcomes, recurrence associations, and tumor-subsite-specific effects [23,38,44,45,46,52].	BMI is a size-for-height measure; it does not quantify visceral fat, skeletal muscle, or nutritional reserves and should not be used as a surrogate for these phenotypes.	Heterogeneous BMI cut-offs; tumor subsite; stage; comorbidity; reverse causality from prediagnostic weight loss.
Visceral/subcutaneous adiposity	Sex-specific adipose-tissue compartments may provide prognostic information beyond BMI; visceral fat appears particularly relevant in men and subcutaneous fat in women [34].	CT-derived adipose distribution is a distinct body-composition phenotype that may complement, but is not interchangeable with, BMI.	Non-standardized thresholds; limited external validation.
Sarcopenia/sarcopenic obesity	Sarcopenia and sarcopenic obesity are associated with postoperative complications and adverse long-term outcomes after gastrectomy [31,32,33].	Skeletal-muscle depletion and sarcopenic obesity are distinct from BMI-defined obesity and may identify vulnerability not apparent from body weight alone.	Definitions and measurement methods vary across studies.
Nutrition/perioperative support	Malnutrition may coexist with overweight or obesity. Current surgical and oncology nutrition guidelines support early oral/enteral feeding and individualized nutritional therapy when nutritional risk is present [29,30].	Nutritional screening and longitudinal weight assessment should complement, rather than be replaced by, BMI classification.	Dietary intake and physical activity are inconsistently assessed.
Sex/hormonal factors	Biological sex, hormonal/reproductive exposures, and sex-specific fat distribution have each been associated with gastric cancer risk or prognosis [34,48,49,50,51,52,53].	These variables should be analyzed separately; observed hormonal associations are hypothesis-generating and do not establish causality.	Sex-stratified analyses are often underpowered; hormonal exposure and adipose distribution may confound or modify observed sex differences.
*Helicobacter pylori*	*H. pylori* is a major etiological determinant, particularly for non-cardia gastric cancer; eradication is associated with reduced gastric cancer risk [8,9].	*H. pylori* status should be considered when interpreting associations of BMI, nutrition, and body composition with gastric cancer.	Incomplete ascertainment or adjustment may cause residual confounding and between-study heterogeneity.
Prediagnostic weight loss/cachexia	Weight loss before diagnosis is associated with poorer survival and can materially alter apparent BMI–outcome associations [54,55,56].	Premorbid weight and longitudinal weight change should be assessed alongside BMI to reduce reverse-causality bias.	Timing and intentionality of weight loss are often unavailable; weight loss may reflect tumor stage, reduced intake, inflammation, or cachexia.

Legend: Conceptual synthesis of the principal domains discussed in this narrative review. BMI, body mass index; CT, computed tomography; *H. pylori*, *Helicobacter pylori*.

**Table 4:** Recent evidence on body weight, body composition, and gastric cancer risk or prognosis.

Study	Design/Population	Key Result	Relevance to the Review	Main Limitation
Azizi et al. [24]	Systematic review and meta-analysis (>14 million participants)	Pooled RR: underweight 1.124 (95% CI 0.968–1.304; not significant), overweight 1.155 (95% CI 1.051–1.270), and obesity 1.218 (95% CI 1.070–1.386). Meta-regression suggested stronger associations for excess body weight in women and non-Asian populations.	Supports an association of excess body weight with gastric cancer risk, while highlighting substantial heterogeneity.	Substantial between-study heterogeneity; observational data; subsite definitions vary.
Pescari et al. [57]	Cross-sectional study (n = 1255 adults)	BMI and body fat percentage (BFP) captured different aspects of obesity; psychological factors predicted obesity by BMI but not by BFP.	Supports complementing BMI with direct body-composition assessment.	Cross-sectional, non-gastric-cancer population; BIA-derived body fat is method-dependent.
Kuichanuan et al. [58]	Observational study in young Thai adults	A revised BMI cut-off was proposed to account for population-specific differences in body composition.	Supports consideration of population-specific BMI thresholds.	Young Thai adults; not a gastric-cancer cohort; limited generalizability.
Luan et al. [56]	Multicenter cohort (n = 29,779 patients with gastric cancer)	Prediagnosis lifestyle analysis included BMI at diagnosis, usual BMI, and weight loss; >10% weight loss was associated with poorer prognosis in the reported analyses.	Supports separating premorbid body size and prediagnostic weight change from BMI measured at diagnosis.	Observational cohort; residual confounding and treatment heterogeneity remain possible.

Legend: Summary of recent studies investigating body weight, body composition, and prediagnostic weight change in relation to gastric cancer risk or prognosis. BMI, body mass index; BFP, body fat percentage; RR, risk ratio.

**Table 5:** BMI, adjuvant chemotherapy, and survival after curative gastrectomy for gastric cancer.

Study	Design/Population	Key Result	Interpretation	Main Limitation
Tsuburaya et al. [65]	Pooled analysis of 3527 patients from randomized trials after D2 gastrectomy	Adjuvant chemotherapy improved relapse-free and overall survival across TN stages. The benefit was greater in T1–2 than T3–4 tumors and was higher in low/intermediate BMI groups than in the high-BMI group (HR 0.58, 0.58, and 1.05).	BMI was associated with heterogeneity in observed treatment benefit, but the analysis does not establish a causal BMI effect.	Post hoc pooled biomarker analysis; BMI subgroup findings do not establish a causal treatment interaction.
Peng et al. [66]	Retrospective cohort of 2510 patients with stage II/III gastric cancer after curative gastrectomy	Low preoperative BMI (<20.3 kg/m^2^) independently predicted incomplete perioperative chemotherapy (<6 cycles) and poorer overall survival (HR 1.317; 95% CI 1.162–1.494; *p* < 0.001). Completion of ≥6 cycles improved survival in low-BMI patients.	Low BMI identifies a vulnerable subgroup in whom treatment completion and nutritional support may be particularly relevant.	Retrospective cohort; chemotherapy completion may mediate or confound the BMI–survival association.

Legend: Summary of studies evaluating the association of BMI with adjuvant chemotherapy and survival after curative gastrectomy. BMI, body mass index; HR, hazard ratio; CI, confidence interval.

## Data Availability

No new data were created or analyzed in this study. Data sharing is not applicable to this article.
